# Orthodontic retreatment using anchorage with miniplate to camouflage a Class III skeletal pattern

**DOI:** 10.1590/2176-9451.21.3.104-115.bbo

**Published:** 2016

**Authors:** Marcel Marchiori Farret

**Affiliations:** 1 Professor, post-graduation courses, Specialization in Orthodontics, Centro de Estudos Odontológicos Meridional (CEOM), Passo Fundo, Rio Grande do Sul, Brazil; and Fundação para Reabilitação das Deformidades Crânio-Faciais (FUNDEF), Lajeado, Rio Grande do Sul, Brazil.

**Keywords:** Angle Class III malocclusion, Tooth extraction, Orthodontic anchorage procedures

## Abstract

This manuscript describes the treatment of a 27-year-old patient who was previously treated with two maxillary first premolar extractions. The patient had skeletal Class III malocclusion, Class III canine relationship, anterior crossbite, and a concave profile. As the patient refused orthognathic surgery, a miniplate was used on the right side of the lower arch as an anchorage unit after the extraction of mandibular first premolars, aiding the retraction of anterior teeth. At the end of treatment, anterior crossbite was corrected, in which first molars and canines were in a Class I relationship, and an excellent intercuspation was reached. Furthermore, patient's profile remarkably improved as a result of mandibular incisor retraction. A 30-month follow-up showed good stability of the results obtained. This case was presented to the Brazilian Board of Orthodontics and Dentofacial Orthopedics (BBO) as one of the requirements to become diplomate by the BBO.

## RESUMO

Esse artigo descreve o caso clínico de um paciente com 27 anos de idade, previamente tratado com exodontias dos primeiros pré-molares superiores. O paciente apresentava padrão esquelético de classe III, relação entre caninos de classe III, mordida cruzada anterior e perfil côncavo. Como o paciente recusou-se a ser submetido a um tratamento ortodôntico-cirúrgico combinado, foi utilizada uma miniplaca no lado direito inferior, após as exodontias dos primeiros pré-molares inferiores, como unidade de ancoragem para a correção da linha média e retração dos dentes anteroinferiores. Ao término do tratamento, a mordida cruzada anterior foi corrigida, os primeiros molares e os caninos estavam em relação de chave de oclusão e uma excelente intercuspidação foi obtida. Além disso, o perfil facial do paciente teve considerável melhora estética, como resultado da retração dos incisivos inferiores. Esse caso foi apresentado à Diretoria do Board Brasileiro de Ortodontia e Ortopedia Facial (BBO), como parte dos requisitos para a obtenção do título de Diplomado pelo BBO.

## INTRODUCTION

This report refers to a patient who sought orthodontic treatment at the age of 27, complaining about his facial and smile esthetics as a result of a concave profile and anterior crossbite. During the first interview, the patient reported he had previously undergone orthodontic treatment during which maxillary first premolars were extracted to allow irruption of maxillary canines. Furthermore, he reported that treatment was only performed in the upper arch. In his medical history, he highlighted a car accident he had suffered a few years before, which was responsible for a scar on the upper lip. 

## DIAGNOSIS

**Figure 1:**
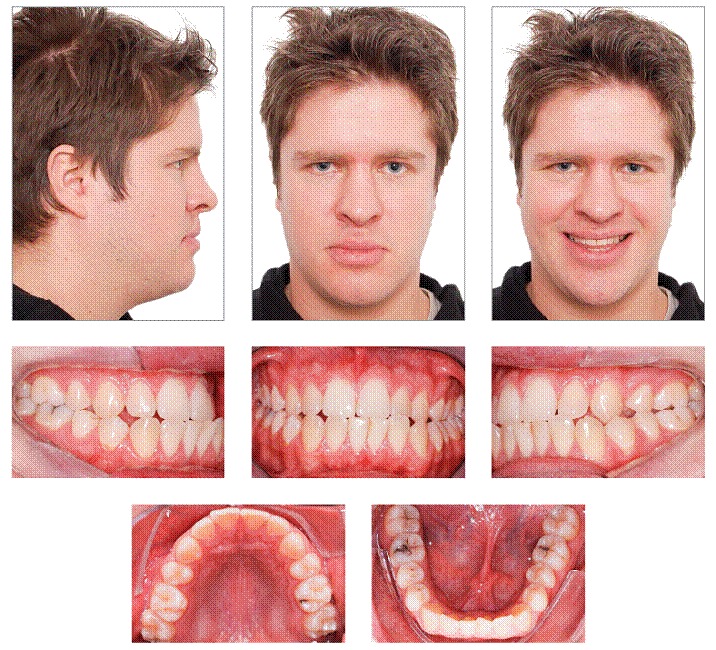
Facial and intraoral initial photographs.

As seen in Figure 1, based on frontal facial analysis, it is clear that there was proportionality among the facial thirds, with no apparent asymmetries. In smile analysis, it was possible to identify reduced maxillary incisors display and anterior crossbite with mandibular incisors proclined with exposition of the tongue. The profile was concave with the lower lip projected, in comparison to the upper lip (upper lip-S Line = −4.5 mm and lower lip-S Line = −0.5 mm). 

Intraoral and dental cast analyses revealed that the patient had Angle Class II malocclusion, subdivision left and Class III canine relationship on both sides. Moreover, he also presented with anterior and posterior crossbite on the left side, lower arch discrepancy of −2 mm, upper midline deviation of 1 mm to the right, and lower midline deviation of 3 mm to the left (Figs 1, ^2^). 

**Figure 2:**
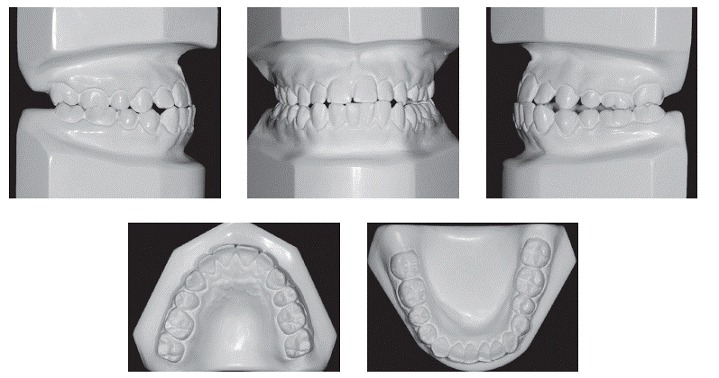
Initial dental casts.

Panoramic radiograph confirmed the absence of maxillary first premolars and all third molars, good parallelism among roots and no root resorption. Cephalometric analysis (Fig 4 and Table 1) revealed Class III skeletal pattern (ANB = −4°), hypodivergent growth pattern (SN.GoGn = 27°, FMA = 16°, and Y-Axis = 53°), and excessive proclination of maxillary (1.NA = 32° and 1-NA = 12 mm) and mandibular incisors (1.NB = 35°, 1-NB = 8 mm, and IMPA = 112°).

**Figure 3:**
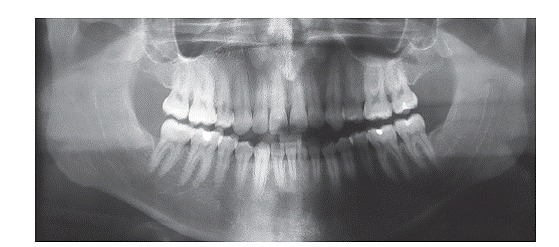
Initial panoramic radiograph.

**Figure 4:**
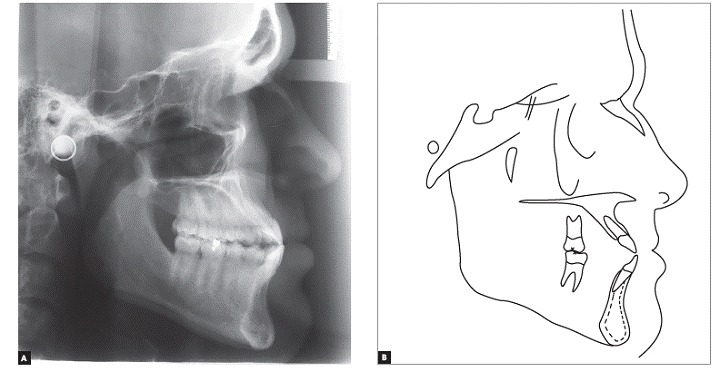
Initial cephalogram (A) and cephalometric tracing (B).

**Table 1:**
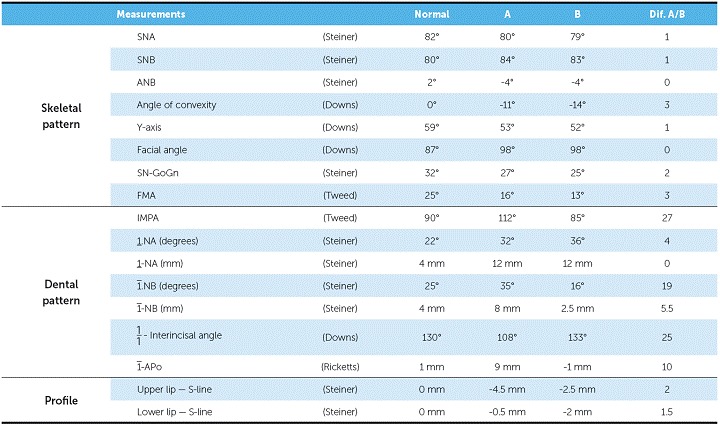
Initial (A) and final (B) cephalometric values.

## TREATMENT PLAN 

Considering the skeletal discrepancy and the concave profile associated with a Class III canine relationship and anterior crossbite, the first treatment option was orthodontic treatment followed by orthognathic surgery for maxillary advancement. However, the patient refused orthognathic surgery and opted to undergo compensatory treatment to camouflage the skeletal problem. Based on the excessive proclination of mandibular incisors, there was a possibility of retraction after the extraction of mandibular first premolars, thereby eliminating anterior crossbite, reducing lower lip projection and improving facial profile esthetics. As the patient had a Class I molar relationship on the right side and a Class II relationship on the left side, with accentuated midline deviation to the left (3 mm), there was a need for great anchorage control on the right side. For this reason, it was considered that a miniplate should be positioned on the external oblique line on the right side, which was accepted by the patient. After miniplate installation, mandibular anterior teeth would be moved to the right side, correcting asymmetries of the lower arch and obtaining a Class I canine relationship. In the upper arch, the insertion of one mini-implant on the left side was planned to correct midline deviation. For retention, after treatment, a 4 × 4 mandibular retainer was bonded to all teeth and was to be used for an undetermined period of time. Additionally, a maxillary removable wraparound retainer was fitted and should be used 24 hours a day for one year, followed by one more year at night only. 

## TREATMENT PROGRESS

Treatment started with the bonding of metallic brackets (Edgewise standard prescription with 0.022 × 0.028-in slots) without torque or angulations on the upper and lower arches, except for mandibular incisors. Alignment and leveling were performed by means of 0.012-in to 0.020-in stainless steel archwires with a bypass in the region of maxillary and mandibular incisors. In the upper arch, the aim of the bypass was to avoid incisor extrusion, which could provoke premature contact due to the edge-to-edge relationship in this region. In the lower arch, the aim of the bypass was to avoid even more proclination of incisors and avoid overload on the wire during masticatory function, which could break the wire in that region. 

At the end of preliminary alignment and leveling, miniplate insertion and mandibular premolars extractions were required. Teeth #46 and #47 were tied together to the miniplate and were to be used as the anchorage unit for distalization of tooth #43 with an elastomeric chain. After partial distalization of tooth #43, mandibular incisors were bonded and the whole arch was aligned and leveled. Subsequently, anterior teeth were retracted with a 0.019 × 0.025-in stainless steel arch with bull loops, and activation on the right side was carried out on the miniplate to avoid any mesial movement of posterior teeth. 

**Figure 5:**
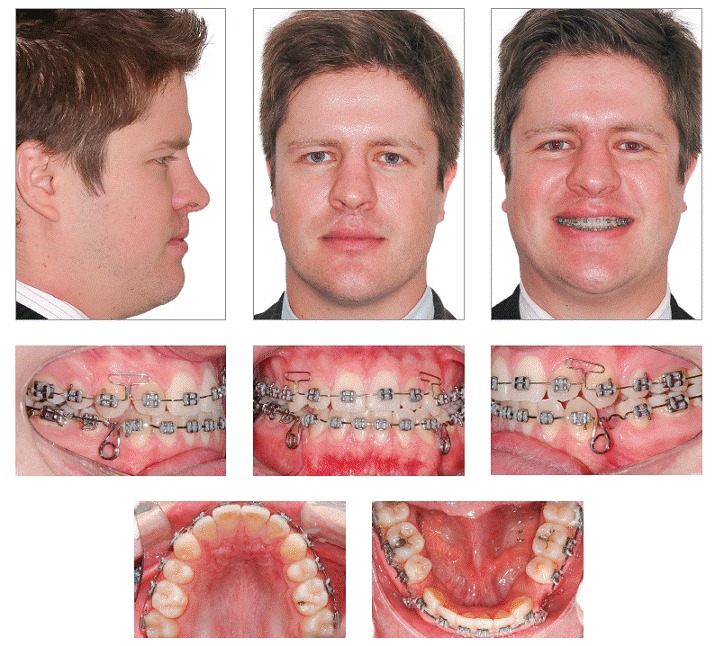
Intermediate facial and intraoral photographs.

After anterior crossbite correction, maxillary incisors were included in the alignment and leveling of maxillary posterior teeth (Fig 5). The mini-implant was inserted between teeth #23 and #24 to correct the upper midline. After upper and lower midline deviation was corrected and a Class I canine relationship was achieved on both sides, the spaces on the left side of the lower arch were closed with elastomeric chains, so as to loosen anchorage. After total closure of spaces, finishing procedures took place. Some brackets were rebonded, and new alignment and leveling were performed on both arches to refine intercuspation.

After verifying that all objectives had been achieved, the devices were debonded and allowed for the retention period to begin. For the upper arch, a removable wraparound appliance was established and the patient was made aware that he had to use it 24 hours a day for the first year and after that for one more year during the night only. For the lower arch, a 4 x 4 retainer was made with a 0.016 × 0.022-in stainless steel piece bonded to all teeth and was to be used for an undetermined period of time. 

## TREATMENT RESULTS

**Figure 6:**
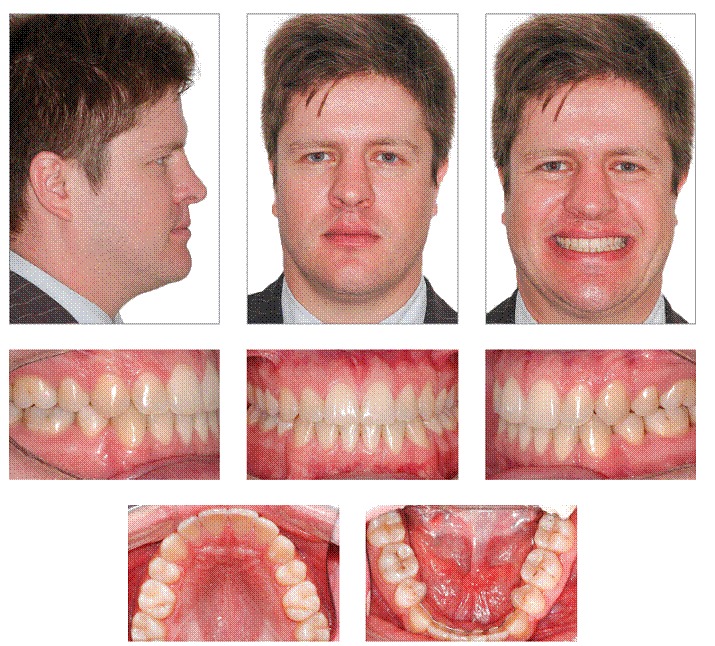
Final facial and intraoral photographs.

By assessing the final records (Figs 6 to ^9^), it is possible to identify that all objectives were achieved. Patient's facial profile showed considerable improvement in esthetics and a harmonic projection between lips. Furthermore, there was remarkable improvement in smile esthetics with anterior crossbite correction, midlines correction, and an increase in maxillary incisor display. In frontal view, there was a balanced face with the mandible well positioned in comparison to the sagittal plane.

**Figure 7:**
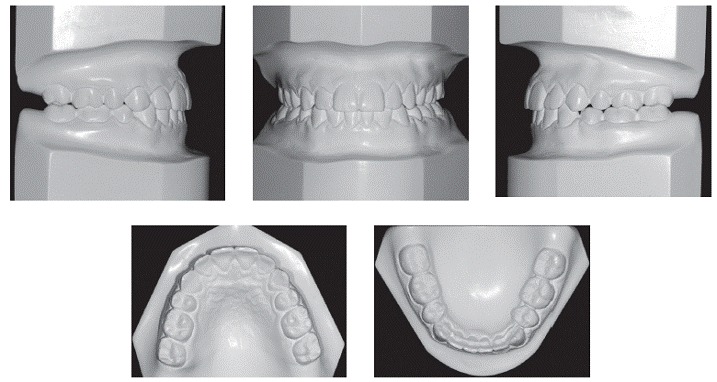
Final dental casts.

**Figure 8:**
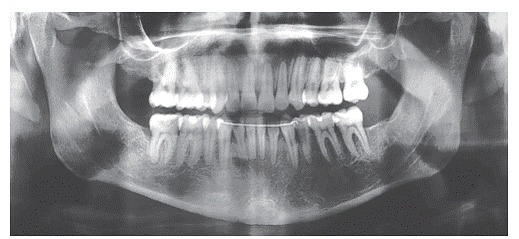
Final panoramic radiograph.

**Figure 9:**
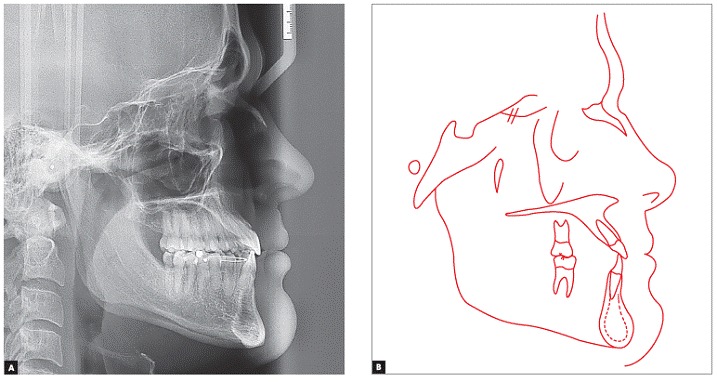
Final cephalogram (A) and cephalometric tracing (B).

**Figure 10:**
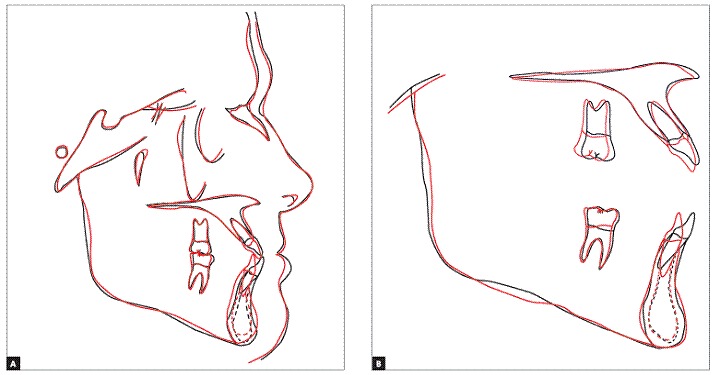
Total superimposition (A), partial superimpositions (B) and initial (black) and final (red) cephalometric tracings.

**Figure 11:**
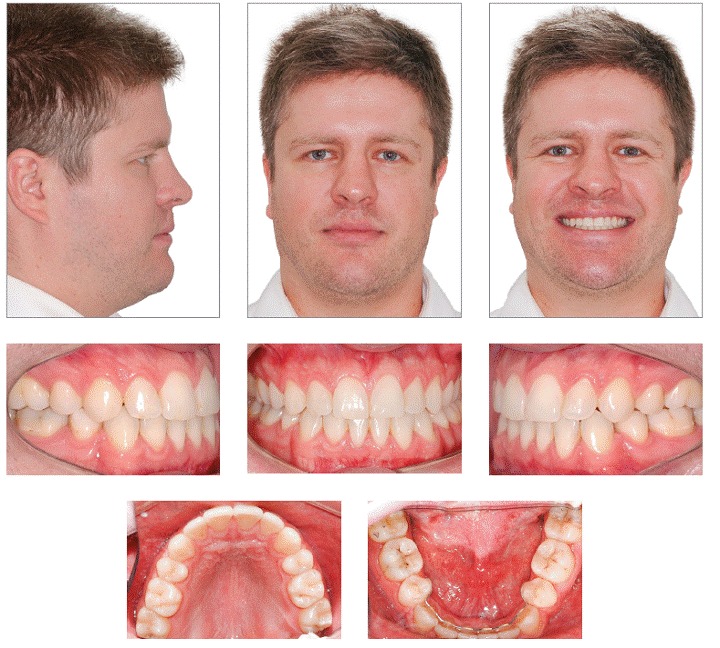
Facial and intraoral photographs of a 30-month follow-up.

Intraoral and dental cast analyses (Figs 6 and ^7^) revealed good alignment and leveling of the arches as well as a Class I relationship for molars and canines. There was anterior crossbite correction, with adequate overjet and overbite. Likewise, it was verified that the midlines were matching, and a good intercuspation was present between maxillary and mandibular teeth, with excellent functional harmony of occlusion either in incisor or canine guidance.

Through panoramic radiograph, it is possible to verify good parallelism among roots and a slight apical remodeling in the roots of maxillary and mandibular incisors (Fig 8). Cephalometric analysis showed that mandibular incisors were remarkably retracted, showing a variation of 19° in 1.NB and therefore became substantially uprighted (1.NB = 16°, 1-NB = 2.5 mm, and IMPA = 85°). There was also expressive improvement in lower lip prominence, which changed from −0.5 mm to −2 mm, thereby resulting in a harmonic relationship with the upper lip. In the 30-month follow-up, we observed excellent occlusal stability with maintenance of the obtained results. 

## FINAL CONSIDERATIONS

In general, skeletal Class III pattern impairs facial esthetics and occlusion. In the reported case, although the best approach would be the association of orthodontics and orthognathic surgery to obtain an excellent esthetic result, it is possible to emphasize that there was remarkable improvement both in esthetics and function, with total patient's satisfaction. In this case, the use of a miniplate was crucial to anchorage control on the right side, which was necessary to achieve asymmetry correction in the lower arch. Furthermore, mandibular incisor projection at the beginning of treatment was determinant for camouflage of the skeletal pattern, as it allows retraction of those teeth, thus eliminating crossbite and obtaining significant response to the lower lip; therefore, balancing the profile. However, it is important to highlight the need for a long-term follow-up procedure to control stability of the results obtained.
